# A Rapid One-Pot Synthesis of Novel High-Purity Methacrylic Phosphonic Acid (PA)-Based Polyhedral Oligomeric Silsesquioxane (POSS) Frameworks via Thiol-Ene Click Reaction

**DOI:** 10.3390/polym9060192

**Published:** 2017-05-27

**Authors:** K. Karuppasamy, K. Prasanna, Dhanasekaran Vikraman, Hyun-Seok Kim, A. Kathalingam, Liviu Mitu, Hee Woo Rhee

**Affiliations:** 1 Division of Electronics and Electrical Engineering, Dongguk University-Seoul, Seoul 04620, Korea; v.j.dhanasekaran@gmail.com; 2 Electrochemical Energy Storage and Conversion Lab (EESC), Kyung Hee University, 1732, Deogyeong-daero, Giheung-gu, Yongin, Gyeonggi 17104, Korea; pras.chemical@gmail.com; 3 Millimeter-wave Innovation Technology (MINT) Research Center, Dongguk University-Seoul, Seoul 04620, Korea; kathu@dongguk.edu; 4 Department of Natural Sciences, University of Pitesti, Pitesti 110040, Romania; ktm7ro@gmail.com; 5 Polymer Materials Lab, Department of Chemical and Biomolecular Engineering, Sogang University, Seoul 04107, Korea

**Keywords:** thiol-ene, POSS, MALDI-TOF, nanomaterials, phosphonic acid

## Abstract

Herein, we demonstrate a facile methodology to synthesis a novel methacrylic phosphonic acid (PA)-functionalized polyhedral oligomeric silsesquioxanes (POSSs) via thiol-ene click reaction using octamercapto thiol-POSS and ethylene glycol methacrylate phosphate (EGMP) monomer. The presence of phosphonic acid moieties and POSS-cage structure in POSS-S-PA was confirmed by Fourier transform infrared (FT-IR) and nuclear magnetic resonance (^1^H, ^29^Si and ^31^P-NMR) analyses. Matrix-assisted laser desorption ionization time-of-flight (MALDI-TOF) mass spectrum of POSS-S-PA acquired in a dithranol matrix, which has specifically designed for intractable polymeric materials. The observed characterization results signposted that novel organo-inorganic hybrid POSS-S-PA would be an efficacious material for fuel cells as a proton exchange membrane and high-temperature applications due to its thermal stability of 380 °C.

## 1. Introduction

In recent years, polyhedral oligomeric silsesquioxanes (POSSs), a family of nanostructured inorganic components with size tunability and functionality, have fascinated global research communities due to their wide range of potential applications in viscosity modifiers, catalysis, coating, drug-delivery vehicles, electronics, and renewable energy sources [1,2,3]. Additionally, it has strong attention due its unique physico-chemical properties and reactive organic terminal functional substituents [4,5,6,7,8]. The three-dimensional structure of the silicon–oxygen (Si–O) core is inevitably responsible for high thermal stability and stiffness, whereas the terminal organic reactive functional groups can be enabled to make the covalent bond between POSSs and polymers, which results in the solid progress of organic–inorganic hybrid materials [9,10]. POSS properties have been tuned [11,12,13,14,15] by incorporating various end-group moieties (functionalities), such as acrylates, methacrylate, alcohols, amines, carboxylic acids, epoxides, fluoroalkyls, halides, and imides, through self-polymerization reactions, which include self-condensing vinyl polymerization, poly-condensation reactions, ring-opening polymerization, and “ene-click” reactions [16,17,18]. Methacrylate-functionalized POSSs provide improved thermal stability and flame retardancy. Further, the incorporation of phosphorus-, silicon-, and boron-containing compounds as POSS moieties is an effective way of improving its physicochemical properties and stiffness [9,19,20,21].

In this communication, we report on the development of a novel type of methacrylic phosphonic acid-functionalized POSSs for fuel cells. To the best of our knowledge, there is no report available based on the synthesis and characterization of methracrylate phosphate-functionalized POSSs (POSS-S-PA). Via thiol-ene click reaction chemistry, octamercapto thiol POSSs were functionalized with 7∼8 phosphonic acid (PA) groups, paving the way for use as a potential candidate for proton exchange membrane fuel cells due to its anhydrous proton conduction group, high thermal, hydrolytic, and oxidative stabilities [21,22].

## 2. Material Synthesis

### 2.1. Materials

2,2′-Azobis(isobutyronitrile) (AIBN) was purchased from Fischer, Seoul, Korea and used after purification. 3-Mercaptopropyl trimethoxysilane (MTS, 98%), anhydrous dichloromethane (DCM), triethyl amine (TEA), and methanol (MeOH, 99.9%) were donated by Sigma Aldrich, Yongin, Korea. Ethylene glycol methacrylate phosphate (EGMP, 98%) was purchased from Shanghai Angewchem Co., Ltd. (Shanghai, China).

### 2.2. Synthesis of POSS-S-PA

**Figure 1:**
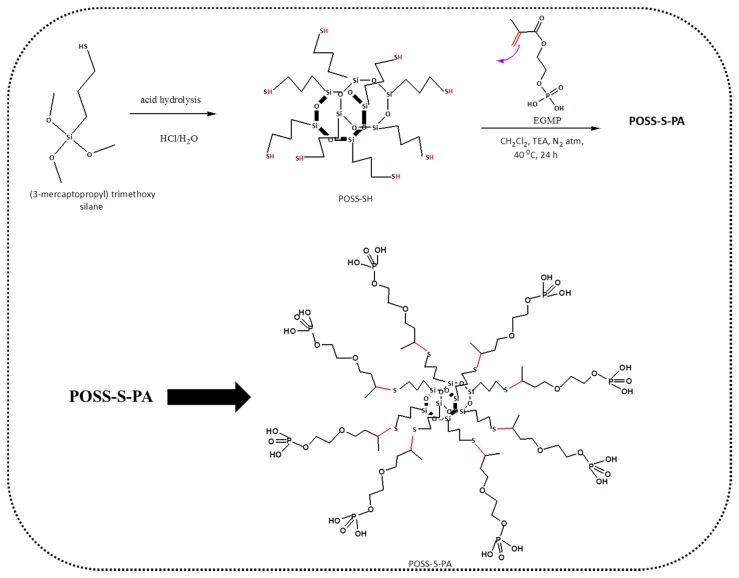
Pictorial representation of thiol-ene click reaction between POSS-SH and EGMP.

The synthesis of POSS-S-PA involves two steps. The first step includes the synthesis of POSS-SH from 3-mercaptopropyl trimethoxysilane by acid hydrolysis reaction [9], which is described as follows. The schematic representation of the synthetic route is sketched in Figure S1 (ESI). Firstly, 15 mL of stoichiometric MTS were mixed with 20 mL of concentrated HCl and 240 mL of an MeOH (anhydrous) solution in a three-neck flask, which was fitted with a condenser, as shown in Figure S1. In order to complete hydrosilylation reaction, the precursor solutions were subjected to acid hydrolysis followed by condensation reactions at 90 °C for 48 h. After the removal of solvent under reduced pressure, the obtained raw product was further dissolved in anhydrous dichloromethane and precipitated in distilled water (18.3 MΩ.cm at 25 °C), and this procedure was repeated three times to obtain the final product. The collected final product as a white-colored viscous liquid with a yield of 22% was dried under vacuum at 80 °C for 12 h. The thiol-ene click reaction between POSS-SH and EGMP was pictorially represented in Figure 1.

The second step for the synthesis of POSS-S-PA from POSS-SH is explained as follows: The methacrylic PA-terminated hybrid-network polymer POSS-S-PA was synthesized by the so-called “thiol-ene” reaction [19,23,24] using POSS-SH and EGMP. For synthesis, 10 mL of POSS-SH (10.1800 g, 1000 mL, and 10 mM) dissolved in 30 mL of anhydrous DCM and TEA (0.24 mM) were taken in a three-necked round bottom flask and subjected to constant stirring under an N_2_ atmosphere for about 2 h at 40 °C. Then, freshly distilled EGMP (16.8080 g, 1000 mL, and 80 mM) was injected into the reaction mixture to carry out the polymerization reaction for 5 h at 40 °C. In order to retain the POSS-cage structure, EGMP was added slowly, dropwise, for up to 30 minutes. The obtained product was kept in a refrigerator overnight, and white precipitate was then separated from DCM using rotatory evaporation. The obtained white precipitate, designated as POSS-S-PA, was further purified by dissolving in methanol and re-precipitation in diethyl ether to remove unreacted POSS-SH, and then filtered and dried. The yield of light yellow POSS-S-PA was 64% and a schematic for the synthetic route is shown in Figure 1. Its corresponding instrumentation details are provided in the supplementary file.

## 3. Results and Discussions

A novel methacrylic POSS-S-PA has been synthesized using octamercapto thiol-POSS and ethylene glycol methacrylate phosphate (EGMP) monomer via thiol-ene click reaction. Further, as-prepared POSS-S-PA was characterized by NMR, FTIR, MALDI-TOF, TG-DTG, and SEM analyses to confirm their hybrid structure.

**Figure 2:**
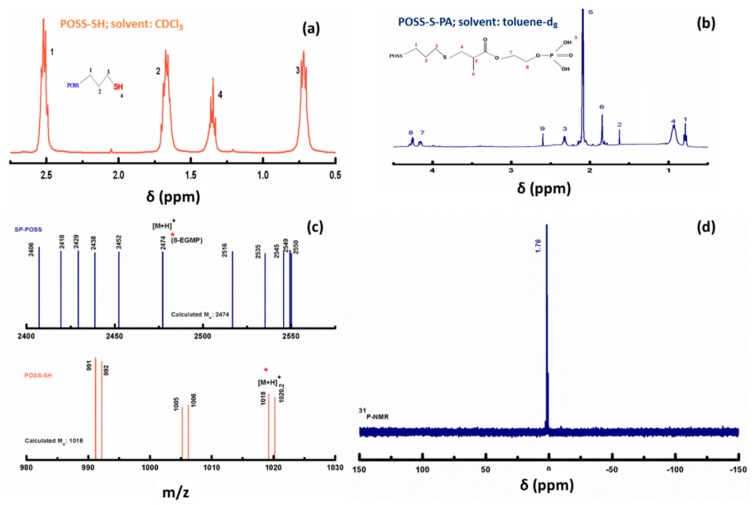
(**a**) ^1^H-NMR spectrum of POSS-SH; (**b**) ^1^H-NMR spectrum of POSS-S-PA; (**c**) MALDI-TOF-MS spectrum of POSS-SH and POSS-S-PA; (**d**) ^31^P-NMR spectrum of POSS-S-PA.

The ^1^H-NMR and ^13^C-NMR spectra of precursor MPTS and product POSS-SH are given in Figure S2 (ESI) which confirms the formation of POSS-SH from MPTS. Figure 2a,b show the ^1^H-NMR spectra of POSS-SH and POSS-S-PA. From the ^1^H NMR spectrum of POSS-SH, three signals are observed at 0.72, 1.68, and 2.52 ppm due to methylene protons on the mercaptopropyl arm and another triplet signal is exhibited at 1.34 ppm, which is attributed to thiol proton. The peak analysis provides an integral of 2:2:2:1 for an eight-armed thiolated POSS peak of 1, 2, 3, and 4, respectively. In POSS-S-PA, the successful completion of reaction between POSS-SH and EGMP has been confirmed by the absence of thiol triplet peak at 1.34 ppm as well as the absence of resonance from the ethylene proton in the range 4.5–6.5 ppm as shown in Figure 2b. The important proton NMR peaks of POSS-S-PA and its assignments are as follows: 0.78 (t, Si–C*H*_2_), 1.62 (s, Si–CH_2_–C*H*_2_), 2.31 (t, Si–CH_2_–CH_2_–C*H*_2_), 0.93 (m, S–C*H*_2_) 2.09 (m, S–CH_2_–C*H*–CH_3_) 1.83 (s, CH–C*H*_3_) 4.14 (t, CO–C*H*_2_), 4.25 (m, –C*H*_2_–P), 2.60 (s, P–O*H*).

Besides, the MALDI-TOF MS spectrum shows two strong signals at 1018 and 2474 m/z for POSS-SH and POSS-S-PA, respectively, as displayed in Figure 2c. These peaks represent the mass of silsesquioxanes (SQ) cage with octamers like eight propyl and EGMP groups, respectively. For the same, the calculated values are 1017 and 2474 *m*/*z*. In addition, some other unassigned signals are observed along with main peaks, but the ultimate product for a building block of the octamer SQ cage is confirmed by elemental and NMR analyses. The unassigned peaks observed may be due to the hexamer and heptamer of the SQ cage, which could not be isolated selectively during the synthesis. The elemental analysis data of both POSS-SH and POSS-S-PA are as follows: POSS-SH: Calculated—C 47.9, H 9.39 and S 42.67; found—C 47.82, H 10.20 and S 42.60%; POSS-S-PA: Calculated—C 45.35, H 6.82, O 33.80 and S 14.03; found—C 45.12, H 6.82, O 33.98 and S 14.08%. These observations are strongly consistent with earlier results [25,26].

**Figure 3:**
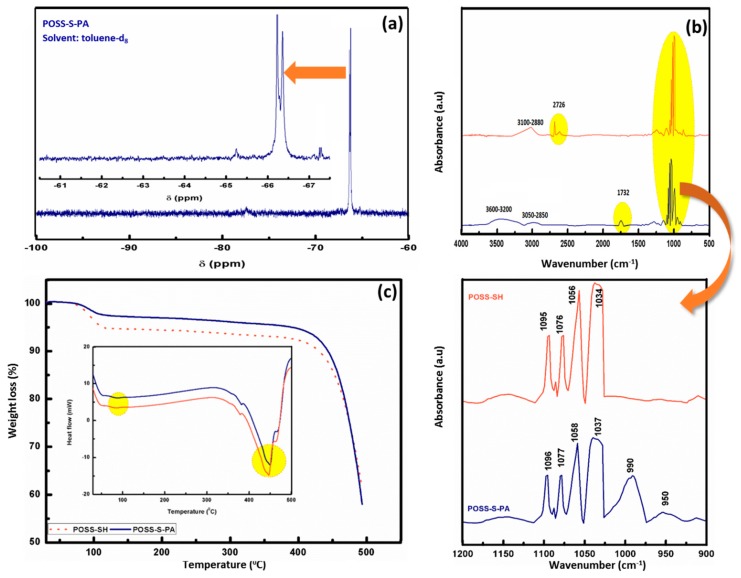
(**a**) ^29^Si-NMR spectrum of POSS-S-PA; (**b**) FT-IR spectra of POSS-SH and POSS-S-PA; (**c**) TG-DTA thermogram of POSS-SH and POSS-S-PA.

The presence of PA terminal groups in the synthesized sample is further confirmed by ^31^P-NMR analysis, and it is displayed in Figure 2d. It shows a sharp signal at 1.6 ppm, which arises due to the diacid form of the phosphonate units in POSS-S-PA. From the ^29^Si NMR spectrum of Figure 3a, two signals are exhibited in the T^3^ region, which confirms the presence of the POSS cage structure. The main signals are observed from −66.10 to −66.38 ppm and from −66.40 to −66.70 ppm and assigned to the cage-like octamer (T^8^) and the cage-like decamer (T^10^), respectively. Moreover, the absence of a silanol group signal in the ^29^Si NMR spectrum of POSS-S-PA confirms the stable SQ cage structure without any degradation during the course of reaction.

FTIR spectroscopy analysis is performed to confirm the various structural organization in POSS-S-PA functional groups as well as to distinguish thiol and double bonds conversion individually [27,28]. Figure 3b represents the attenuated total reflection (ATR)–FT-IR spectra of POSS-SH and POSS-S-PA hybrids. The observed peaks are at 1100–1080 and 2626 cm^−1^ attributed to characteristic Si–O–Si stretching and S–H stretching vibrations of POSS-SH, respectively [29,30]. The *thiol-ene* click reaction formed POSS-S-PA is confirmed by the presence of C=O, C–H stretching and P–OH peaks are at 1732, 2850–3050, and 3600 cm^−1^, respectively, as well as the absence of an S–H peak in the region of 2620 cm^−1^. The observed FTIR peaks and its corresponding assignments of POSS-SH and POSS-S-PA are tabulated in Table 1. The observed results are highly consistent with earlier literature based on the thiol-methacrylate system [31,32].

**Figure 4:**
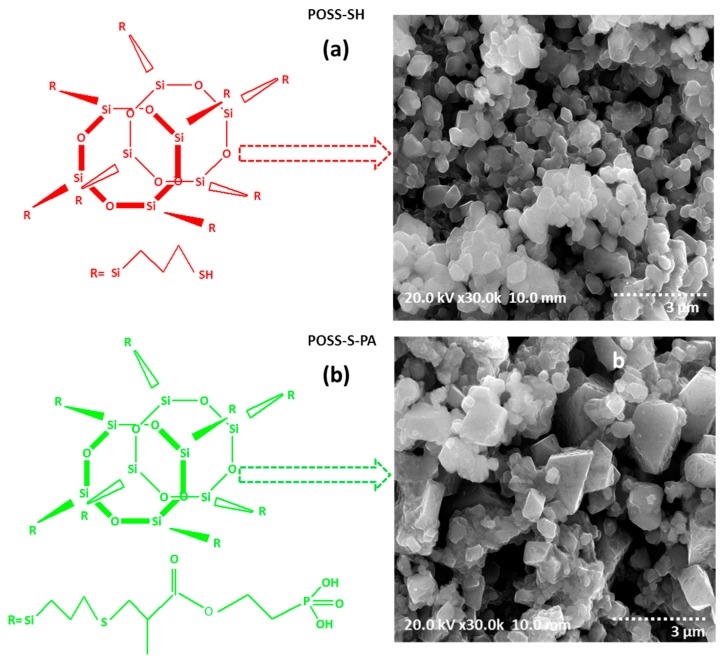
Structure and SEM image of (**a**) POSS-SH and (**b**) POSS-S-PA hybrids.

TG-DTA curves of POSS-SH and POSS-S-PA hybrids are provided in Figure 3c. From the curves, the weight loss is observed for less than 5% up to 120 °C, which may be due to the volatilization of free and hydrogen bonded water; thereafter, the continuous weight loss is observed until 434 °C, attributed to the decomposition of terminal groups in the POSS core and the complete decomposition of POSS backbones. The related DTA curve with respect to heat flow also shows a similar type of thermal variation during the entire weight loss curve, which is given in inset of Figure 3c. The morphological properties of POSS-SH and POSS-S-PA hybrids were investigated by FE-SEM analysis (Figure 4a,b). It can be seen from the SEM micrograph that POSS-SH consists of a 3D-skeletal like morphology, with a combination of spherical and hexagonal shaped grains. For POSS-S-PA (Figure 4b), the SEM image consists of larger-sized grains due to the agglomeration.

## 4. Conclusions

In summary, a novel strategy of a PA-functionalized SQ cage was successfully prepared with high yields by a rapid one-pot synthesis via thiol-ene click reaction. The excellent purity of the as-prepared POSS-S-PA framework was confirmed by NMR, FTIR, and MALDI spectral analyses. Our hybrid structure of POSS-S-PA possessed good thermal stability, which was exposed by TG-DTA. From the observed results, POSS-S-PA would be an excellent inorganic–organic hybrid material for high-temperature applications and as a proton exchange membrane in fuel cells to help to increase the protonic transport, which in turn improves its protonic conductivity and cell performance.

## Figures and Tables

**Table 1:** Peak positions and its corresponding assignments of POSS-SH and POSS-S-PA.

FTIR Frequency Wavenumber (cm^−1^)		Assignments
POSS-SH	POSS-S-PA	
1095–1056	1096–1058	Si–O–Si
-	990, 950	P–O stretching & P–C stretching
-	1736	C=O
2690	-	S–H
3100–2880	3050–2850	C–H stretching
-	3200–3600	P–OH stretching

## Supplementary Materials

The following are available online at www.mdpi.com/2073-4360/9/6/192/s1, Figure S1: Schematic diagram for acid hydrolysis of 3-mercaptopropyl trimethoxysilane; Figure S2: ^1^H and ^13^C- NMR spectra of of 3-mercaptopropyl trimethoxysilane and POSS-SH.
