# Supplementary material for: A Rapid One-Pot Synthesis of Novel High-Purity Methacrylic Phosphonic Acid (PA)-Based Polyhedral Oligomeric Silsesquioxane (POSS) Frameworks via Thiol-Ene Click Reaction
Source: Polymers (Basel). 2017 May 27;9(6):192. doi: 10.3390/polym9060192 (PMC6432363; doi:10.3390/polym9060192)
Supplement: Supplementary file 1 [file polymers-09-00192-s001.pdf]

Supplementary Materials: A Rapid One-Pot Synthesis of Novel High-Purity Methacrylic Phosphonic Acid (PA)-Based Polyhedral Oligomeric Silsesquioxane (POSS) Frameworks via Thiol-Ene Click Reaction

K. Karuppasamy, K. Prasanna, Dhanasekaran Vikraman, Hyun-Seok Kim, A. Kathalingam, Liviu Mitu and Hee Woo Rhee

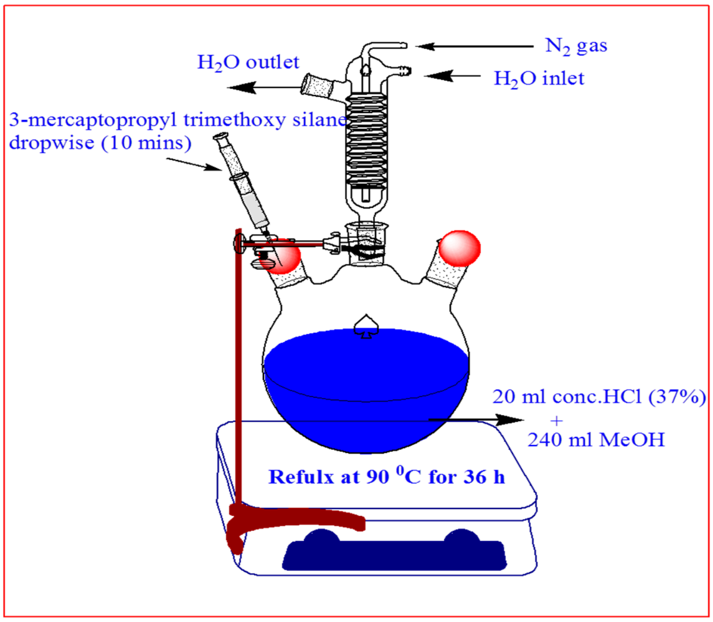

Figure S1. Schematic diagram for acid hydrolysis of 3-mercaptopropyl trimethoxysilane.

Instrumentation

^{1}H-NMR, ^{31}P-NMR spectra of all synthesized hybrids were taken using $CDCl_3$ and toluene-d_8 solvent by Bruker avance (500 MHz) AV400 spectrometer. A ^{29}Si spectrum was recorded using a JEOL ECX-400 spectrometer using toluene-d_8 as solvent. Chemical shift values in ppm were referenced to TMS for ^{1}H-NMR spectra. FTIR spectra were recorded with the help of a Nicolet 380 FT-IR spectrometer (Thermo Electron) in the region 4000-400 cm^{-1} at a signal resolution of 1 cm^{-1}. Elemental analyses were carried out on an Elemental Vario Micro Cube elemental analyzer. The matrix assisted laser desorption ionization time of flight mass (MALDI TOF-MS) analysis was carried out with the help of Bruker Autoflex Speed Series mass spectrometer (Bruker Daltonics, Leipzig, Germany) using dithranol, Silver trifluoroacetate (AgTFA) and chloroform were used as matrix, cationizating agent, and solvent, respectively, under positive ion mode. The thermogravimetry (TGA) analysis of prepared materials was carried out on a TGA-2950 thermal analyzer (Hi-Res, TA instruments) by heating from 25 to 700 °C under a N_2 atmosphere at a heating rate of 20 °C/min. Before TGA experiment, an isothermal segment was carried out under nitrogen atmosphere at 120 °C for 30 min. The morphology analysis of POSS-SH and POSS-S-PA were investigated by field emission scanning electron microscopy (FE-SEM) JSM-6700F.

^{1}H-NMR and ^{13}C NMR analyses

The various important proton and carbon NMR peaks of 3-mercapto trimethoxysilane and POSS-SH were assigned and represented in Figure S2.

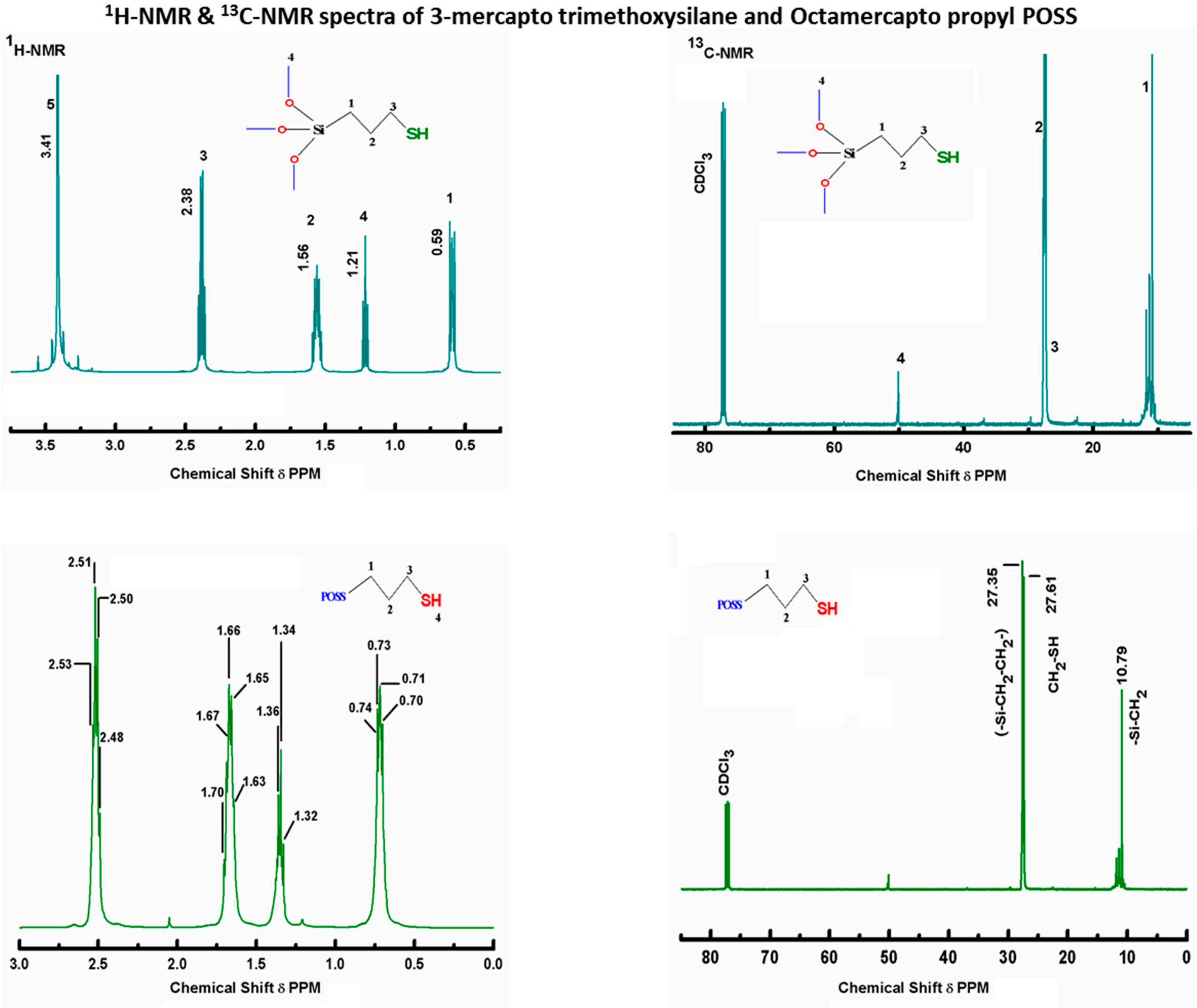

Figure S2. ^{1}H and ^{13}C-NMR spectra of of 3-mercaptopropyl trimethoxysilane and POSS-SH.

[1] Kotal, A.; Si, S.; Paira, T.K.; Mandal, T.K. Synthesis of Semitelechelic POSS-Polymethacrylate Hybrids by Thiol-Mediated Controlled Radical Polymerization with Unusual Thermal Behaviors. *J. Polym. Sci. Part A Polym. Chem.* **2008**, *46*, 1111–1123; doi:10.1002/pola.22453.
